# Myeloproliferative disorder associated with alopecia universalis

**DOI:** 10.1016/j.jdcr.2024.07.007

**Published:** 2024-07-27

**Authors:** Mardeen S. Karim, Heeran S. Karim, Pranita V. Rambhatla

**Affiliations:** a Department of Dermatology, Henry Ford Health, Detroit, Michigan; b Department of Biology, University of Virginia, Charlottesville, Virginia

**Keywords:** alopecia areata, alopecia universalis, essential thrombocythemia, Janus kinase, myeloproliferative disorder, oncodermatology, ruxolitinib

## Introduction

Alopecia areata (AA) is a T lymphocyte-mediated autoimmune disorder of nonscarring hair loss with narrowing of the hair shaft near the scalp, clinically.^1^^,^^2^ Histologically, AA is characterized by hair follicle bulbs surrounded by infiltrating T cells.^1^, ^2^, ^3^ The progression of AA is uncertain, exhibiting diverse durations and extents of the condition. Typically, alopecia presents as a couple patches affecting the scalp, eyebrows, eyelashes, or body hair. However, in severe instances, individuals may experience complete hair loss on the scalp, known as alopecia totalis, or loss of all scalp and body hair, referred to as alopecia universalis (AU). The predominant pathophysiologic theory for AA primarily focuses on the breakdown of immune privilege within the hair follicle; 2 primary theories elucidate the initial events: 1 theory highlights localized abnormalities in hair follicles, whereas the other implicates an immune system dysfunction.^1^ Loss of immune privilege leads to an immune response, encompassed by activation of the Janus kinase/signal transducer of activation (JAK/STAT) pathway, which has been a major focus in studies of the pathogenesis and treatment of AA. JAK represents the downstream signaling pathway triggered by interferon gamma and interleukin 15, pivotal players in AA induction.^1^ Activation of JAK redirects CD8^+^ T cells toward cytotoxic roles against the hair follicle bulb regardless of T-cell receptor involvement.^1^ In this report, we describe a case of AU as a consequence of aberrant JAK/STAT signaling in a patient with myeloproliferative disorder (MPD) and how treatment with a JAK/STAT inhibitor led to significant clinical hair regrowth.

## Case report

**Fig 1:**
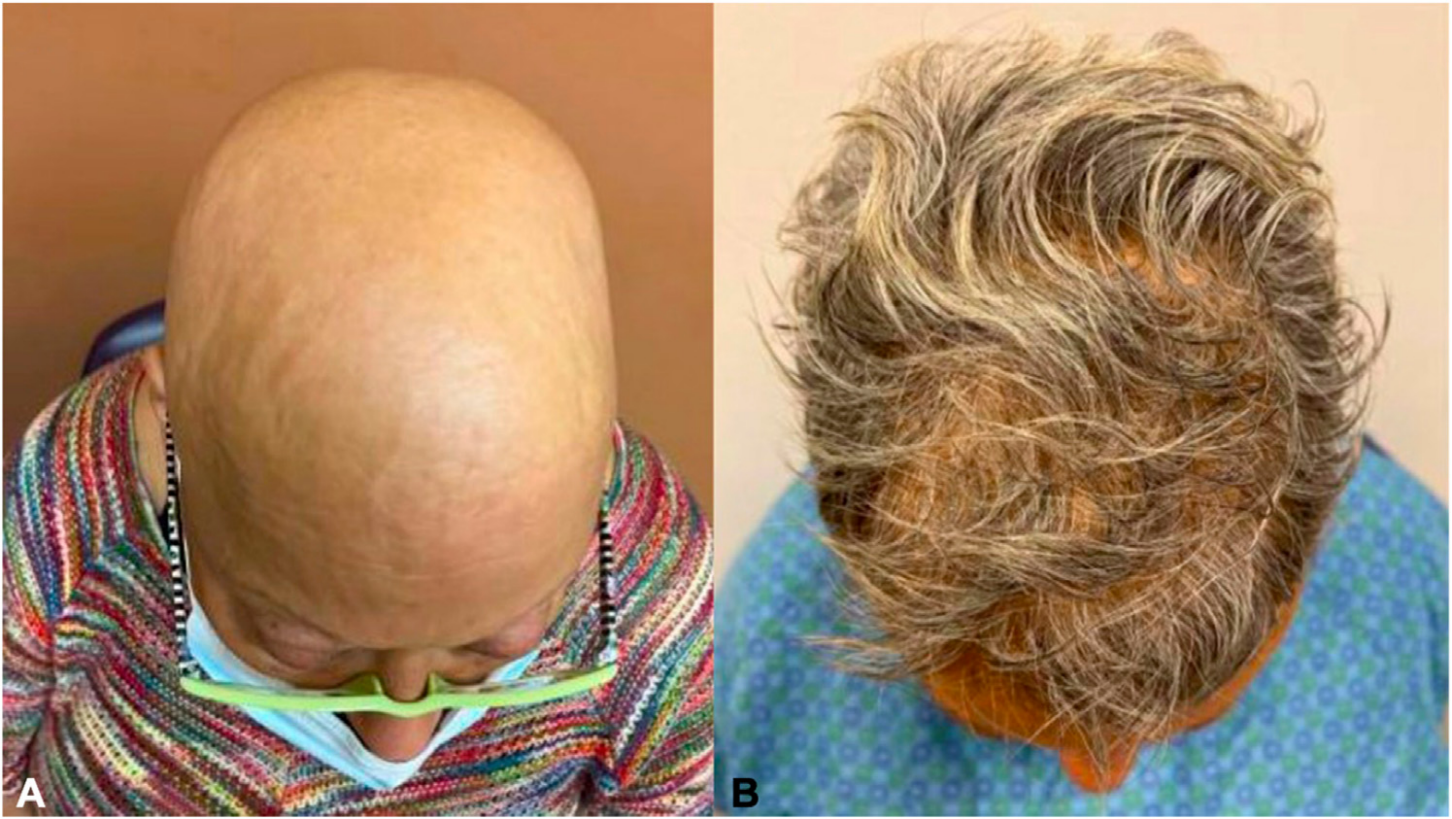
**A,** Total alopecia of the scalp in a patient with myeloproliferative disorder. **B,** Robust hair growth on the scalp after 4 months of treatment with ruxolitinib.

A 67-year-old woman with a history of JAK2-V617F–positive MPD and essential thrombocythemia (ET) presented to dermatology clinic with a 9-month history of complete hair loss. Her MPD was diagnosed via bone marrow biopsy 1.5 years before presentation and was not refractory to treatment with infrequent courses of hydroxyurea and consistent therapeutic phlebotomy. Physical examination demonstrated total alopecia of the scalp, eyelashes, eyebrows, axillae, and extremities (Fig 1, *A*). She was diagnosed with AU. The patient’s MPD had been attempted to be managed with hydroxyurea however given waves of thrombocytopenia due related comorbidities including chronic gastrointestinal bleeding and gastric arteriovenous malformations, all requiring hospital admissions and procedures, the patient was not able to be maintained on a steady treatment dose. After discussion with her hematologist, hydroxyurea was discontinued indefinitely and ruxolitinib 10 mg twice daily was initiated in an attempt to treat both AU and MPD. After 4 months of treatment, the patient experienced robust hair growth on the scalp (Fig 1, *B*). The patient also reported notable hair growth of her eyebrows, eyelashes, genitalia, axillae, and bilateral upper and lower extremities. While on therapy, the patient did not necessitate therapeutic phlebotomy.

## Discussion

MPDs represent a group of genetic disorders of hematologic stem cells leading to the overproduction of mature red blood cells, white blood cells, and/or platelets.^3^^,^^4^ Specifically, ET is a MPD presenting with persistent platelet count elevation accompanied with possible thrombosis and hemorrhage.^4^ Although patients with ET can be afflicted by a broad set of symptoms—including headache, respiratory symptoms, nausea, and nonspecific bone pain—associated hair loss has rarely been reported.^4^ In this context, we present a rare case involving ET complicated by AU.

Malfunctioning of the JAK/STAT pathway leads to 2 primary categories of diseases: immunomodulatory disorders and neoplastic conditions, predominantly hematologic in nature.^5^ The presence of JAK2-V617F mutation in MPDs, including ET, contributes to upregulated JAK-STAT signaling.^3^^,^^4^ Ruxolitinib, an inhibitor of the JAK1 and JAK2 protein kinases, is approved for treatment in myelofibrosis.^2^, ^3^, ^4^, ^5^ Similarly, JAK-STAT signaling pathways have been trialed as a therapeutic target in AA with baricitinib recently becoming the first Food and Drug Administration–approved treatment for adults with severe AA. Prior studies have well established the cytolytic effect of autoreactive T cells on hair follicles in AA.^1^, ^2^, ^3^, ^4^ Additionally, studies have shown increased serum inflammatory cytokines in patients with ET, enhanced T helper 17 cell development with STAT3 activation, and chronic activation of antigen-presenting cells in the context of JAK-STAT signaling.^1^^,^^3^ These examples highlight that upregulation of JAK-STAT pathways can set the stage for autoimmune processes, such as AA.

One can hypothesize that the upregulated inflammation associated with constitutive activation of the JAK-STAT pathway can be associated with the pathogenesis of AA/AU. However, very few known reports have linked AA as a consequence of the aberrant signaling in MPD.^3^^,^^4^ This poses the questions: is this a common, underrecognized phenomenon where individuals with AU have underlying, undiagnosed MPD, or is this a rare phenomenon where a complex interplay of genetic, environmental, and immunologic factors make individuals with diagnosed MPD prone to AU? The latter hypothesis could explain why individuals with JAK2-mutated MPDs are not commonly reported to have alopecia although 50% to 60% of patients with MPDs have the associated mutation.^6^ Further research is needed to address these questions and elucidate pathophysiologic mechanisms. Regardless, MPDs should be considered in patients presenting with significant hair loss in the context of hematologic abnormalities.

## Conflicts of interest

None disclosed.
